# A New Technique for Solving Tightrope Cutout during Acromioclavicular Joint Fixation: A Case Report

**DOI:** 10.5704/MOJ.1703.003

**Published:** 2017-03

**Authors:** BW Ng, AF Abdullah, S Nadarajah

**Affiliations:** Department of Orthopaedics, Melaka Hospital, Melaka, Malaysia

**Keywords:** TightRope cutout, clavicle fracture, ACJ disruption, TightRope on tubular plate

## Abstract

Acromioclavicular joint (ACJ) dissociation is one of the common injuries affecting adults. The stability of ACJ largely depends on the integrity of acromioclavicular ligament, coracoclavicular ligament, capsule, trapezius muscle and deltoid muscle. The injury has been classified by Rockwood into six types and treatment options can be guided by the classification. TightRope fixation is one of the many surgical procedures available to address acromioclavicular joint separation. It consists of tensioning of a no. 5 Fibrewire suture secured at both ends to low-profile metallic buttons. Despite various advantages of using this technique, complications such as suture cut-out, clavicle fracture and suture failure have been documented. The author presents a case of a type III acromioclavicular joint dissociation treated with TightRope which suture cutout was noted intra-operatively. Decision to amend the fixation using a cut one-third tubular plate as an additional anchor for the metallic button on the clavicle was made. Patient’s progress was evaluated using the University of California at Los Angeles Shoulder Score (UCLA Shoulder Score) and significant improvement was noted six months post operatively. We propose this technique as a solution to the encountered problem.

## Introduction

Acromioclavicular joint (ACJ) dissociation is one of the common injuries affecting adults^1-3^. The stability of ACJ largely depends on the integrity of acromioclavicular ligaments, coracoclavicular ligaments, joint capsule, trapezius and deltoid muscles^1^. The injury has been classified by Rockwood into six types and treatment options can be guided according to type of injury sustained^1-3^.

Fixation with TightRope suture button (Naples, Florida, USA) is one of the many surgical procedures available to address acromioclavicular separation^1,2^. It consists of tensioning of a no. 5 Fibrewire suture secured at both ends to low-profile metallic buttons^1,2^. Despite various advantages of using this technique, complications such as suture cut-out, clavicle fracture and suture failure have been documented.

## Case Report

**Fig. 1:**
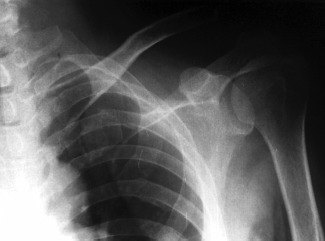
Type III Acromioclavicular joint dissociation.

A 43 years old male motorcyclist presented on day one after a motor vehicle accident. He complained of left shoulder pain and inability to fully abduct and flex his left arm. On examination, there was asymmetry of left shoulder contour as compared to right shoulder. There was tenderness on palpation over his left acromioclavicular joint. Forward flexion of left arm was from 0 to 90 degrees and abduction of left arm was up to 90 degrees. No neurological deficit noted. Radiograph of the left shoulder AP view showed a type III acromioclavicular joint dissociation. The patient was diagnosed to have an acute acromioclavicular joint dissociation (Rockwood Classification Type III). Open acromioclavicular joint reconstruction using TightRope was planned with the aim of reduction of the ACJ dissociation (Fig. 1).

The procedure was performed in beach chair position under general anaesthesia. A longitudinal incision was made over the left acromioclavicular joint extending down to the left coracoid process. The coracoclavicular joint was reduced with a reduction clamp. A tunnel was created through the left clavicle into the coracoid process using a guide pin and cannulated drill. TightRope was then passed through the tunnel. Intra-operatively, cutout of the clavicle during tensioning of tightrope was noted, resulting in a fracture of anterior cortex of left clavicle. A decision to maintain the reduction using a one-third tubular plate as an additional anchor to the tightrope system was made.

**Fig. 2:**
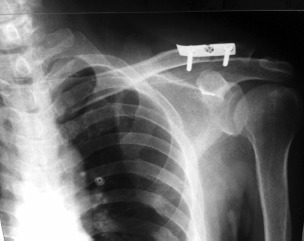
Radiograph showing reduced acromioclavicular joint. TightRope was supplemented with one-third tubular plate as additional anchor.

A three-hole one-third tubular plate was fixed on the clavicle using two cortical screws. The proximal metallic button of the tightrope was anchored above the plate through a small cut made on the middle hole of the tubular plate (Fig. 2). The TightRope was then tightened with the plate functioning as an additional anchor. Postoperatively patient was put on an arm sling and started on passive range of motion exercises. Trapezius and deltoid strengthening exercises were started at 4th week after the procedure. Patient was allowed active full range of motion after 6th week follow-up.

The patient’s progress was evaluated at six month and scored 29/35 using the University of California at Los Angeles Shoulder Score (UCLA Shoulder Score). Active forward flexion of left shoulder was measured to be from 0 to 110 degrees with good muscle strength. Patient did not require analgesics for daily activities.

## Discussion

There are few reports in the literature on complications of the TightRope procedure. Woodmass *et al* reported pooled rate of fracture following arthroscopic fixation of acromioclavicular joint disruption as 5.3% with majority of fractures involving the coracoid process^4^. Kany *et al *reported a case of clavicle fracture due to multiple attempts in clavicle tunneling during the TightRope procedure and it was salvaged using a locking plate. However, exact technique of salvage procedure was not discussed^5^. The incomplete clavicle fracture in this case report is likely due to poor positioning of guide pin and multiple attempts in drilling through the clavicle leading to tunnel widening. It is therefore necessary to ensure proper tunnel position and good reduction is achieved prior to tensioning of the TightRope.

Similar technique can be found in fixation of fibula fracture with a concomitant syndesmotic joint disruption of ankle. To date, no reports have described the use of such technique in dealing with cutout of TightRope causing incomplete clavicle fracture. We suggest the use of this technique if suture cut-out is encountered during this procedure.
